# Distinct root‐associated fungal communities may account for the persistence of *Podocarpus* conifers in tropical forest

**DOI:** 10.1002/ecy.70519

**Published:** 2026-09-25

**Authors:** Astrid Ferrer, Blaine Martin, Adriana Corrales, James W. Dalling

**Affiliations:** ^1^ Department of Plant Biology University of Illinois Urbana‐Champaign Illinois USA; ^2^ Society for the Protection of Underground Networks Dover Delaware USA; ^3^ Smithsonian Tropical Research Institute Panama City Republic of Panama

**Keywords:** Fortuna, Glomerales, Helotiales, Leotiomycetes, mycorrhizae, Panama, root microsite, Trechisporales

## Abstract

Podocarps (Podocarpaceae) are one of the few lineages of conifers found in closed canopy tropical forests. They typically have highly restricted distributions that are associated with nutrient‐poor sites. Unlike other tree species, the presence of distinct nodule‐like structures allows their roots to be easily distinguished from those of co‐occurring taxa. In highly infertile ever‐wet lower montane forest at Fortuna, Panama, roots of *Podocarpus oleifolius* occur in three microsites: in the soil; lying on top of surface litter; as aerial roots that climb the trunks of neighboring trees. We examined how microsite affects *Podocarpus* root fungal community composition by sequencing the internal transcribed spacer (ITS) region, and by measuring root nutrient concentration and phosphatase enzyme activity. We also compared *Podocarpus* roots collected from the soil with those of two common co‐occurring angiosperm trees, *Graffenrieda bella* and *Weinmannia pinnata*. We found that root microsite accounted for 11% of the variation in root‐associated fungal communities and also affected root calcium, potassium, and manganese concentration. Root fungal diversity, phosphate concentration, and phosphatase activity were unchanged. Across all microsites, the fungal order Helotiales accounted for 50%–60% of ITS reads, while aerial roots had significantly higher abundance of Trechisporales. When comparing across species, Glomeromycota were only detected in very low numbers overall (1.8%–3.3% of reads in angiosperms), and were especially low in *Podocarpus* (0.14% of reads). In contrast, *Podocarpus* had a higher fraction of reads belonging to Leotiomycetes than *Graffenrieda* and *Weinmannia*. Our findings highlight how root‐associated fungal communities vary at the order level with microsite and host species in tropical montane forest. In continuously cool, low‐nutrient wet environments, where nutrients are rapidly leached from throughfall, or are mineralized slowly from decomposing organic matter, alternatives to arbuscular mycorrhizal fungi may be especially advantageous to plants. Biotrophic fungi in the Helotiales have been implicated in nutrient transfer in alpine and arctic environments, and together with Trechisporales, may be key unrecognized mutualistic partners in the tropics. Collectively, these taxa may explain the association of podocarps with low‐nutrient montane forest.

## INTRODUCTION

The evolution of flowering plants led to the entire reassembly of tropical plant communities and major feedbacks on global climate (Boyce & Lee, 2010). The dominant plants that preceded the angiosperms (conifers, cycads, lycopods) largely disappeared from the modern day tropics. However, one conifer family, the Podocarpaceae (“podocarps”; 185 species, Farjon, 2001), that evolved 220 million years ago (MYA) (Strullu‐Derrien et al., 2018) successfully recolonized tropical forests 60 MYA and remain a consistent, albeit rare, component of tropical montane forests worldwide (Morley, 2011). The ability of podocarps to compete successfully with broad‐leaved angiosperms in tropical and southern temperate forests has been attributed to modifications in the coniferous needle structure, where flattening increased efficiency of light capture beneath a forest canopy (Brodribb, 2011). However, podocarps share another consistent feature: the presence of nodule‐like structures on roots, a trait shared with the sister family Araucariaceae (Escapa & Catalano, 2013). The function of these root structures as yet lacks a generally accepted explanation (Dickie & Holdaway, 2011). Early studies suggested that nodules house nitrogen (N)‐fixing bacteria (Bond, 1967), but subsequent work either failed to demonstrate N‐fixation (Baylis, 1969; Ushio et al., 2017), or found only low activity (Huang et al., 2007). However, one study of the podocarp *Lepidothamnus fonkii* from a bog in southern Patagonia reports that N fixation can occur in the dead outer cells of nodules by free living diazotrophic bacteria (Borken et al., 2016).

The first descriptions and anatomical drawings of podocarp roots and nodules also highlighted the presence of fungi (Spratt, 1912) with characteristics of arbuscular mycorrhizal (AM) taxa (Becking, 1965). The presence of root nodules, and the general association of podocarps with nutrient‐poor or ultramafic‐derived soils (Cernusak et al., 2011) suggests that their root morphology and associated root microbiomes might confer a resource acquisition advantage under conditions of low soil fertility. In temperate forests of New Zealand, podocarps are associated with phosphorus (P) impoverished terrace and moraine habitats, avoiding fern dominated alluvial sites (Coomes et al., 2005; Russell et al., 2002). Similar associations are found with infertile tropical soils. For example, in lowland Amazonia, podocarps are only reported from N‐ and P‐deficient white‐sand forests near Iquitos, Peru. Podocarps occur more frequently in neotropical montane forests but can also show highly restricted distributions. For example, *Podocarpus oleifolius* occurs on rhyolitic quartz‐rich soils in western Panama and Costa Rica; *Podocarpus selowii* occurs on quartz sands in coastal Atlantic forest, Brazil; *Podocarpus tepuiensis* is restricted to ancient sandstone mountain tops in Guyana, Venezuela, and Ecuador (Dalling et al., 2011).

To date, relatively few studies have analyzed podocarp roots to identify their associated fungi, or to compare them with coexisting angiosperm communities. Unlike Pinaceae, Podocarpaceae do not appear to form ectomycorrhizal (EM) associations. Russell et al. (2002) found extensive arbuscule structures typical of Glomales in the central cortical cells of nodules of *Dacrycarpus* and *Dacrydium* podocarps from New Zealand. Sequencing with fungal internal transcribed spacer (ITS) and Glomales‐specific primers uncovered a number of *Glomus* and *Archaeospora* and two ascomycete taxa. Similarly, Wubet et al. (2006) used glomeromycota‐specific primers to identify lineages of AM fungi and found 16 *Glomus*, three *Diversispora*, and one *Archaeospora* sequence types from *Podocarpus falcatus* from dry Afromontane forest in Ethiopia. Deeper Illumina MiSeq sequencing using primers that target fungal ITS has revealed much higher fungal diversity including both Glomeromycota and Ascomycota in podocarp seedlings from Mount Kinabalu in Borneo (Ushio et al., 2017). Community analyses showed that different podocarp species largely shared both the same AM fungi and non‐AM fungal communities, but these differed from angiosperms collected in the same forest plot. High diversity and read number for ascomycete fungi among the podocarp taxa roots suggest that endophytic fungal groups other than AM fungi or EM fungi may potentially play a role in nutrient uptake in these trees.

**FIGURE 1:**
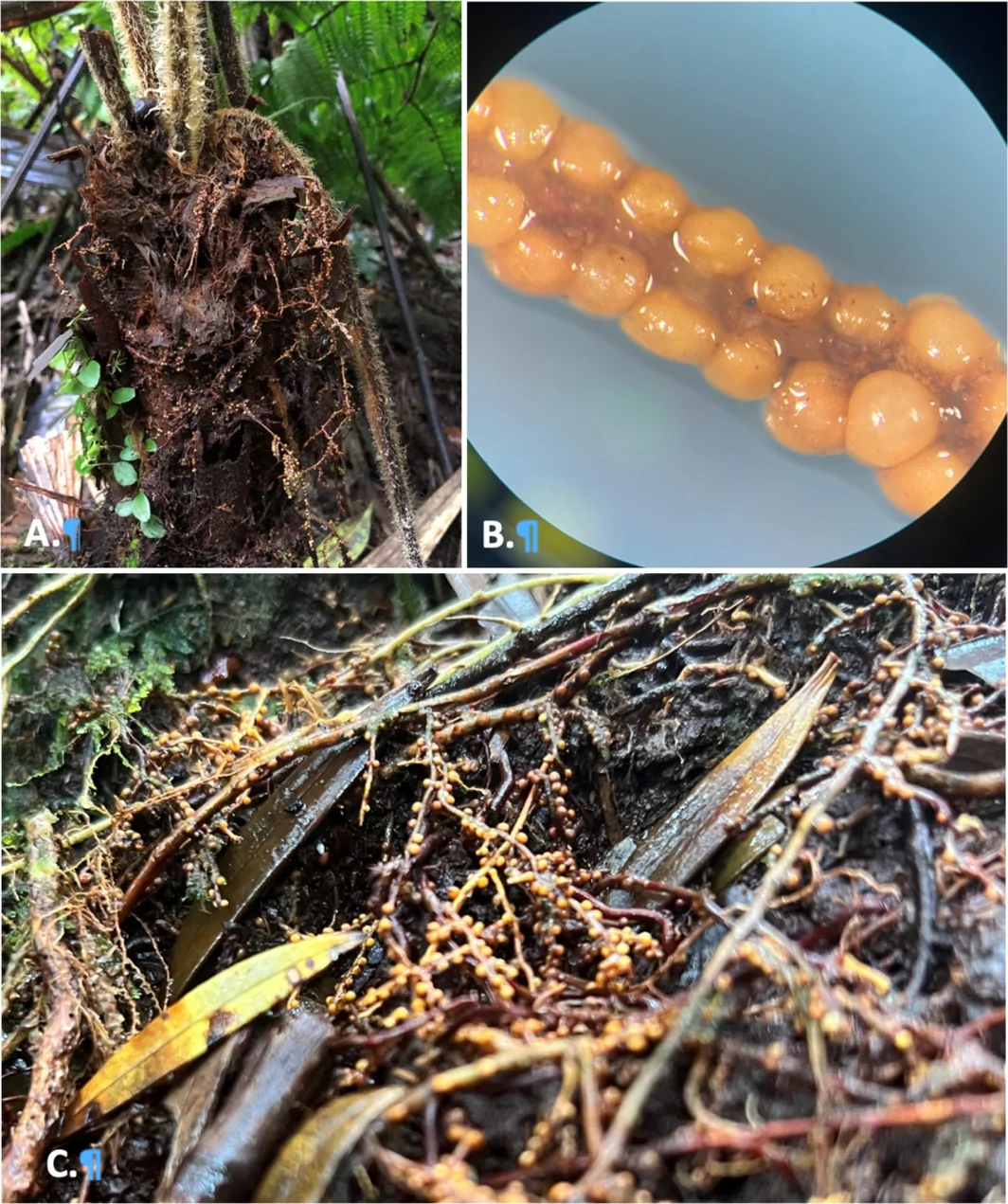
(A) Aerial roots of *Podocarpus oleifolius* covering the stem apex of a tree fern in the Chorro watershed, Fortuna, Panama; (B) close‐up of *P. oleifolius* nodule structure; (C) *P. oleifolius* litter surface roots. Photograph credits: James Dalling (A, C); Blaine Martin (B).

Here we use a combination of fungal primers to characterize root‐associated fungal assemblages of *P. oleifolius*, a canopy tree found in exceptionally wet and infertile sites in the Chorro watershed of the Fortuna Forest Reserve in western Panama. An additional notable feature of *P. oleifolius* at Chorro is the presence of surface roots that lie on top of the litter layer, and aerial roots that climb from the soil onto neighboring trees. Because of the unmistakable nodulated morphology of podocarp roots, it is possible to trace those roots, often extending >5 m up the trunks of trees (Figure 1). Roots in these different microsites are likely to encounter distinct nutrient environments and microbial communities. We predicted that: (1) Roots growing in aerial and litter surface microsites would have lower fungal diversity, fewer AM fungi taxa, and distinct fungal communities than roots in the soil, reflecting different fungal colonization potential and nutrient pools across microsites. (2) Differences in nutrient availability would result in differences in root chemistry and phosphatase enzyme activity across microsites. Finally, the more general association of podocarps with nutrient‐impoverished soils and their distinct root morphology suggest that they may form associations with fungi that are distinct from those of co‐occurring angiosperms. We therefore used an existing sequence dataset from Chorro to compare fungal communities of podocarp roots collected from the soil microsite with those of two common co‐occurring angiosperm taxa, *Weinmannia pinnata* and *Graffenrieda bella*, commonly found in wet montane neotropical forests.

## METHODS

### Study sites and species

The study was based at the Smithsonian Tropical Research Institute Jorge Arauz field station in western Panama. Sample collection was made at the Chorro watershed in the Fortuna Forest Reserve (8° 44′ 55″ N, 82° 13′ 46″ W, 1100 m elevation). Chorro is a premontane super humid rainforest (Holdridge et al., 1971) receiving 5500 mm rain annually, and >95% cloud cover year‐round (Dalling et al., 2021). There is a very shallow organic soil layer only a few centimeters deep, underlain by a deep deposit of silica‐rich rhyolite tuff resulting in highly infertile soil with low N and base cation availability (Dalling et al., 2021). A clay‐enriched subsurface soil horizon impedes drainage with redoximorphic features indicating anaerobic conditions at 40 cm soil depth (Turner & Dalling, 2021). The forest canopy is dominated by palms (*Colpothrinax aphanopetala*, *Euterpe precatoria*, *Wettinia quinaria*) accounting for 33% of stems. The understory is dominated by a tree fern, *Cyathea rojasiana*, endemic to Fortuna (Dalling et al., 2021).

*Podocarpus oleifolius* has a wide distribution from southern Mexico, Central America, and the Andes to Bolivia. At Fortuna, it has a highly restricted distribution occurring on rhyolite substrates at Chorro and in the adjacent Honda watersheds (Dalling et al., 2021). At Chorro, *Podocarpus* trees attain large sizes (>60 cm dbh) and account for 7% of plot basal area. *Weinmannia pinnata* (Cunoniaceae) is common at Chorro, has a similarly wide geographic distribution to *Podocarpus*, and is also restricted to rhyolite soils at Fortuna (Dalling et al., 2021). *Graffenrieda bella* (Melastomataceae) is a Chorro specialist taxon, endemic to Panama. It is among the most abundant tree species in the watershed, accounting for 8% of basal area (Dalling et al., 2021).

### Root collection and imaging

Roots of *Podocarpus* were collected from five adult trees in February 2020. Roots were easily recognized because of the presence conspicuous nodule‐like structures (Figure 1). For each tree, three replicate root sections were collected from three microsite types: (1) “soil” roots that were collected by digging into the mineral soil layer; (2) “litter” roots that were present on the surface of the soil litter layer; (3) “aerial” roots that were collected from the above‐ground surfaces of trees and palms. In 2016, roots were collected from the soil from 24 individuals including seedlings (<20 cm tall), saplings (~20–50 cm tall), and adult trees of each of *W. pinnata* and *G. bella* present at the same Chorro site (i.e., within 100 m of our *Podocarpus* trees). For these two species, roots were excavated from the trunk of each individual until fine roots that were clearly connected to the tree were found. Roots were thoroughly washed with tap water to removed surface soil particles, with a final rinse using sterile water. Approximately, 20 cm of fine roots were preserved in 2% cetrimonium bromide, and then frozen at −20C until processed for DNA extraction.

To visualize fungal colonization of *Podocarpus* roots and nodules, a sample of soil roots were rinsed, cleared in 10% potassium hydroxide solution, and stained using wheat germ agglutinin conjugated with Alexa Fluor 488 to label fungal hyphae and chitin‐rich structures. Stained roots were imaged using a Zeiss Laser Scanning Microscope 880 Airyscan confocal microscope (full methods in Appendix S1).

### DNA extraction and gene amplicon sequencing

Two separate DNA extractions and amplicon sequencing datasets were combined for this study. For *Podocarpus*, root samples were cut into small fragments and DNA extracted from approximately 0.5 g (±0.25 g) of root material using the Qiagen DNeasy PowerPlant Pro DNA extraction kit. Extraction followed the manufacturer's protocol; negative controls for the Pro DNA extraction kit and the polymerase chain reaction were included. The fungal ITS2 was sequenced with primers fITS7 and ITS4 (Ihrmark et al., 2012), and the fungal ribosomal large subunit (LSU) was sequenced using the primers LR0R‐LR3 (Amend et al., 2010; Vilgalys & Hester, 1990). Tagged amplicons were obtained simultaneously using the Fluidigm Access Array system (Fluidigm Corp; San Francisco, CA, USA). Sequencing was performed using the Illumina NOVASEQ 6000 (2×250 paired‐end reads; Illumina Inc., San Diego, CA, USA) at the W.M. Keck Center (Urbana, IL, USA). For *Weinmannia* and *Graffenrieda*, initial root processing followed the same protocol, with DNA extracted using the Mo Bio PowerSoil DNA isolation kit. The LSU amplicons were obtained with the LR0R‐LR3 primers using Fluidigm as above, and sequencing performed using Illumina MiSeq (2×250 paired‐end reads).

Raw Illumina fastq files were demultiplexed by the sequencing center. We used *cutadapt* v. 3.7 to remove primer from all sequence reads (Martin, 2011). To identify amplicon sequence variants (ASVs), data were processed with the R package DADA2 (version 4.10.2). ASVs were independently inferred from the forward and reverse reads of each sample using run‐specific error rates, and then read pairs were merged (Callahan et al., 2016). For the ITS sequences, ASVs were grouped into operational taxonomic units (OTUs) based on a 97% similarity threshold with the DECIPHER package (version 2.30) (Wright, 2016). For LSU, DADA2 was run separately for sequencing datasets using the forward reads, and ASVs from both runs were then merged and grouped into OTUs using the 99% similarity threshold with DECIPHER. Data were imported into QIIME2 (version 2024.10). OTUs with fewer than 10 reads across the entire dataset were removed. Taxonomic annotation was performed for the ITS using the Unite database version 10 and for the LSU using the NCBI BLAST suite, as described by Wahl et al. (2018).

### Root trait measurements

*Podocarpus* root phosphatase (phosphomonoesterase) was measured on ~500 mg of fresh fine root (three replicate root sections per root microsite per tree for the five trees) following the method of Batterman et al. (2018). Root samples were incubated for 30 min at 26°C at pH 5 with para nitrophenyl (*p*NP) phosphate as a buffer. Product (*p*NP) was measured spectrophotometrically at 405 nm with appropriate blanks (no roots) and controls (no substrate). Enzyme activity is expressed as micromoles of para nitrophenyl per gram of root dry mass per hour (μmol *p*NP g^−1^root dry mass h^−1^).

The same number of samples were analyzed for elemental chemistry. Root carbon and N concentrations were measured by grinding 10 mg of root tissue followed by combustion in a Costech 4010 Elemental Analyzer. For all other elements fine root samples were dry‐ashed at 550°C for 1 h in a muffle furnace and the ash dissolved in 1 M nitric acid. Base cations and P were measured using inductively coupled plasma‐ optical emission spectrometry (ICP‐OES) on an Optima 2000 DV (Perkin Elmer, Waltham, MA, USA).

### Data analysis

Analyses were conducted with version 4.1.3 of R (R core team, 2022). For ITS sequence data (*Podocarpus* root microsite data), OTU diversity and richness estimates were made using the full sequence dataset. Shannon diversity and Chao richness estimates (Chao & Chiu, 2016) were calculated using the *vegan* package in R (Oksanen et al., 2022). Shannon diversity values were compared across microsites using a general linear model with the package *lme4* (Bates et al., 2015) with root sample within microsite as a random effect. To compare compositional variation among root microsites, only OTUs that were present in three or more samples were included. Removal of these OTUs excluded taxa that could appear to be microsite specialists because of their rarity and ensured that OTU accumulation curves reached an asymptote. Compositional variation of fungal communities was visualized using three fitted axes in nonmetric multidimensional scaling (NMDS) ordinations of the Morisita–Horn dissimilarity index using the function metaMDS with square root transformation and Wisconsin double transformation. We used permutational analysis of variance (PERMANOVA) to assess the significance and variation explained by microsite on OTU composition using the adonis2 function in *vegan*. To determine whether significant PERMANOVA results were due to shifts in community composition or potentially resulted from differences in community heterogeneity, the “betadisper” function was used to compare microsites. Differences in root nutrient concentration and phosphatase activity were analyzed using a linear mixed‐effects model with tree as a random effect; comparisons among root microsites were made using Tukey honestly significant difference, with Kenward‐Rogers corrected degrees of freedom using the *emmeans* package (Lenth & Piaskowski, 2026).

For LSU analyses (comparisons of *Podocarpus*, *Graffenrieda* and *Weinmannia*) we first excluded samples with <1000 reads and *Podocarpus* samples from litter and aerial microsites. We then pooled sequence data by tree for *Podocarpus* to account for more limited spatial variation in sample location for tree versus seedling roots (in *Graffenrieda* and *Weinmannia*). The remaining dataset consisted of 5 *Podocarpus* samples, and 24 samples of each of *Graffenrieda* and *Weinmannia*. Ordination (NMDS) and PERMANOVA were performed as above.

Finally, we used the classification of OTUs to compare the read fractions present in different taxonomic groups. For ITS data of *Podocarpus* root microsites, we compared the four most abundant orders: Helotiales, Chaetothyriales, Trechisporales, and Russulales, using mixed‐effects general linear models with tree as a random effect and quasi‐binomial errors implemented using the *lme4* package. For LSU data comparing host species, we first compared read numbers across the three most abundant phyla (Ascomycota, Basidiomycota, and Glomeromycota), then across the four most abundant classes (Sordariomycetes, Leotiomycetes, Agaricomycetes, and Dothideomycetes), then across the four most abundant orders (Helotiales, Agaricales, Trechisporales, and Sebacinales). Quasi‐binomial errors were used in cases where Gaussian errors did not meet model assumptions.

## RESULTS

### Richness, diversity, and composition of fungi across root structures and root microsites

**FIGURE 2:**
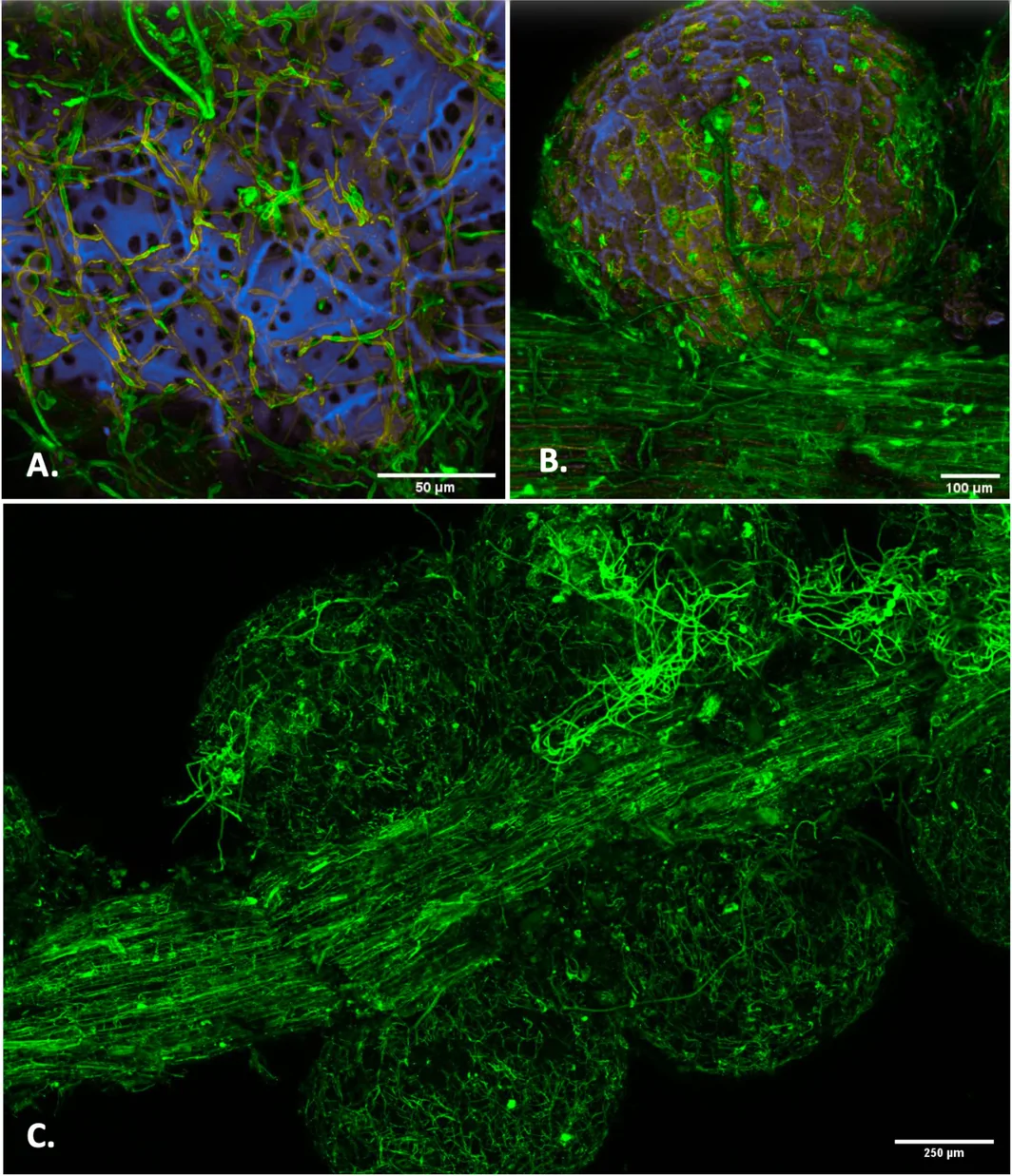
Visualization of fungal colonization and root structure in *Podocarpus oleifolius* nodules using confocal imagery. (A) Z‐stacked confocal image of the root nodule surface showing fungal hyphae and chitin‐rich structures labeled with WGA–AF488 (green) and host tissues with propidium iodide (PI) (blue). Scale = 50 μm. (B) Z‐stacked confocal image of the root nodule interface using the same stains. Scale bar = 100 μm. (C) Tile‐scan of multiple nodules along a lateral root stained with WGA–AF488 to show fungal distribution. Scale bar = 250 μm. Roots were cleared in 10% potassium hydroxide and imaged with a Zeiss Laser Scanning Microscope 880 Airyscan confocal microscope (excitation/emission: WGA–AF488, 488/500–550 nm; PI, 535/617 nm). Credit for all images: Blaine Martin.

*Podocarpus* roots, including both nodules and cortical tissue, were very heavily colonized by fungal structures, including septate and aseptate hyphae, vesicles, and arbuscules (Figure 2). In total, for the ITS region we obtained 18,926,828 sequence reads, representing 1384 OTUs for *Podocarpus* roots collected across the three rooting microsites. Species accumulation curves showed that OTU richness was higher for litter surface roots, than soil roots, with intermediate richness for aerial roots (Table 1, Appendix S1: Figure S1). Shannon OTU diversity was not significantly different across the three root microsites for *Podocarpus* (general linear model: df 2, χ^2^ = 1.49, *p* = 0.47). Overall, 191 OTUs (14%) were in common across the three microsites, and 25%–28% across pairs of microsites.

**TABLE 1:** Internal transcribed spacer (ITS) sequence reads, OTUs and Chao estimated OTU richness (SE in parentheses) for *Podocarpus* roots collected from three microsites.

Rooting microsite	Observed OTU	Chao estimated OTU richness	Shannon diversity	Samples (*n*)
Aerial	686	1452 (105)	2.29	15
Litter	784	1660 (111)	2.20	15
Soil	586	1062 (73)	2.41	14
All	1384	2453 (108)	2.30	44

Abbreviation: OTU, operational taxonomic unit.

**FIGURE 3:**
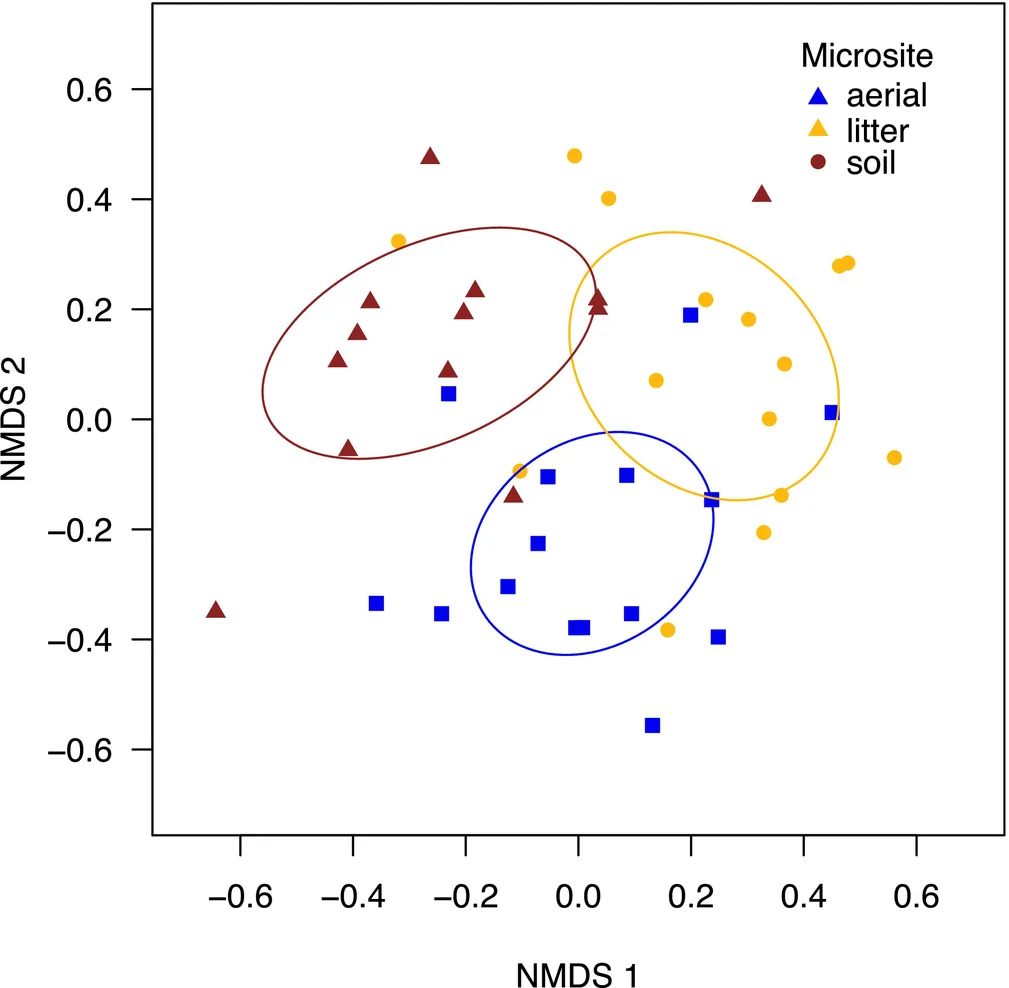
First two axes of a nonmetric multidimensional scaling (NMDS) ordination (*k* = 3, stress = 0.195) representing similarity in fungal communities associated with roots of *Podocarpus oleifolius* sampled from three microsites. Ellipses represent 1 SD of point scores relative to the microsite centroid.

There was a significant effect of rooting microsite (aerial, litter, soil) on fungal communities accounting for 11.1% of compositional variation (Permanova, df 2,41, *F* = 1.51, *p* < 0.001; Figure 3). Post hoc comparisons showed significant differences between all three root categories (*p* < 0.05). Multivariate dispersions were similar among the root microsite groups (Betadisper test, df 2,41, *F* = 0.83, *p* = 0.44).

**FIGURE 4:**
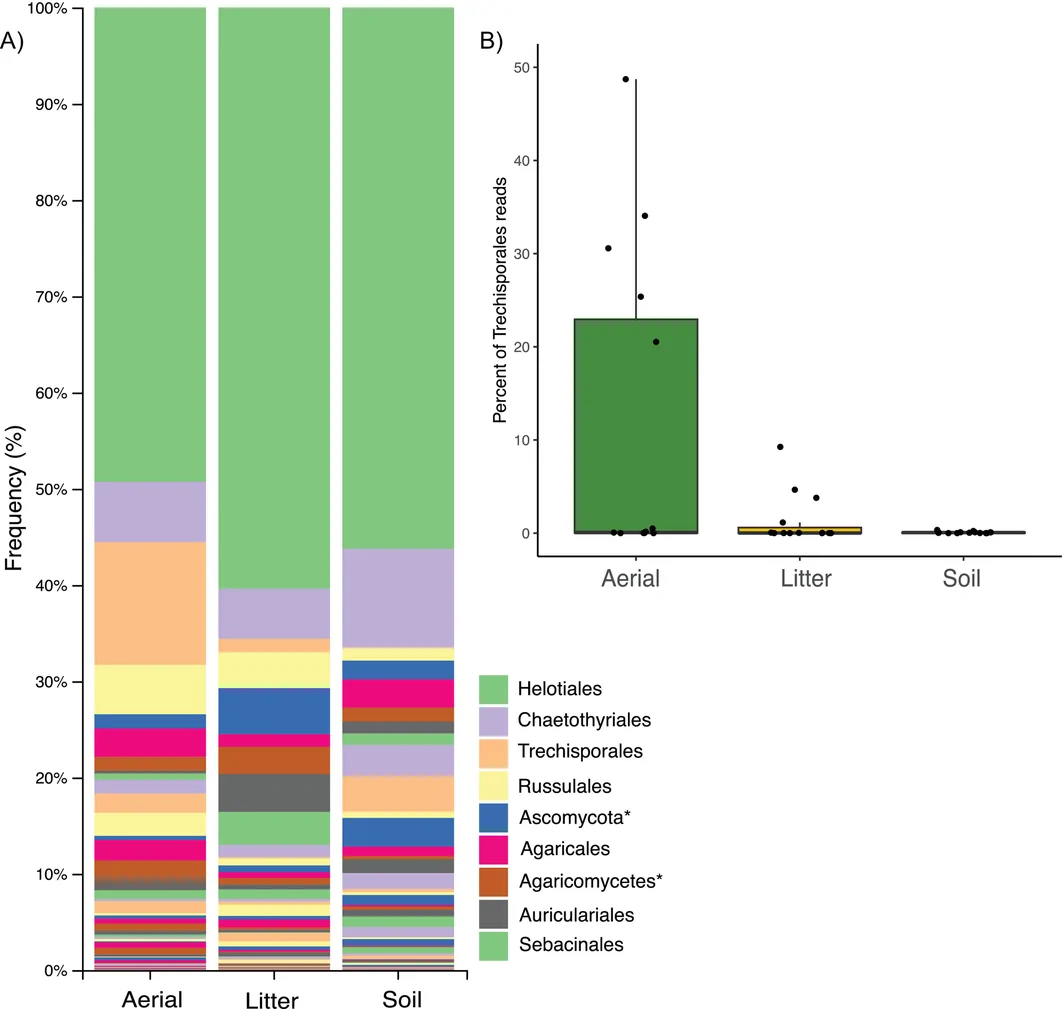
(A) Distribution of internal transcribed spacer (ITS) reads across fungal orders for aerial, litter, and soil root microsites. The top nine orders are named in order of appearance in the aerial root microsite. (B) Percent of reads assigned to the four most abundant orders that were attributed to Trechisporales.* indicates taxa that are *incerta sedis*.

Overall, we found that *Podocarpus* roots were dominated by fungi in the order Helotiales, accounting for ~50%–60% of all ITS reads in each of the root microsites (Figure 4A). The four most abundant orders accounted for ~70%–75% of reads. Among these orders, only Trechisporales differed significantly in read abundance among root microsites (Figure 4B; general linear model df 2, χ^2^ = 10.4, *p* < 0.01) with significantly higher abundance in aerial roots than in litter and soil roots.

### Root nutrient concentration and enzyme activity

Roots sampled in different microsites differed in nutrient concentration (Table 2). Concentrations were generally higher for litter and soil microsites than for aerial roots. Differences were particularly notable for Ca, K, and Mn. In addition, root Al concentration was higher in soil than in litter and aerial roots. Microsite differences were not significant for root C:N and P concentrations. There was no significant difference in root phosphatase activity among microsites (df 2, χ^2^ = 5.24, *p* = 0.07), although aerial roots tended to have lower activity (103.9 ± 39.1 μmol g^−1^ h^−1^ mean ± 1SD) than litter (132.0 ± 12.0 μmol g^−1^ h^−1^) and soil roots (139.3 ± 41.2 μmol g^−1^ h^−1^).

**TABLE 2:** Mean mineral nutrient concentration (1 SE) for roots collected from different microsites.

Element	Aerial	Litter	Soil	χ^2^	df	*p*
Al	0.05 (0.05)^a^	0.05 (0.05)^a^	0.21 (0.18)^b^	18.67	2	<0.001
Ca	0.23 (0.13)^a^	0.37 (0.23)^a,b^	0.51 (0.22)^b^	16.05	2	<0.001
K	0.11 (0.05)^a^	0.20 (0.10)^b^	0.18 (0.09)^b^	11.37	2	0.003
Mg	0.10 (0.05)^a^	0.17 (0.06)^b^	0.14 (0.05)^b^	13.61	2	0.001
Mn	0.02 (0.01)	0.04 (0.04)	0.05 (0.05)	4.93	2	0.111
C:N	31.4 (4.7)^a^	31.7 (4.6)^a^	28.2 (2.5)^a^	7.30	2	0.026
P	0.04 (0.01)^a^	0.05 (0.01)^a^	0.05 (0.01)^a^	10.97	2	0.004

*Note*: Values are micrograms per gram except for C:N. Letters indicate significant differences between microsites (*p* < 0.05).

### Comparison of *Podocarpus* with *Graffenrieda* and *Weinmannia*

**FIGURE 5:**
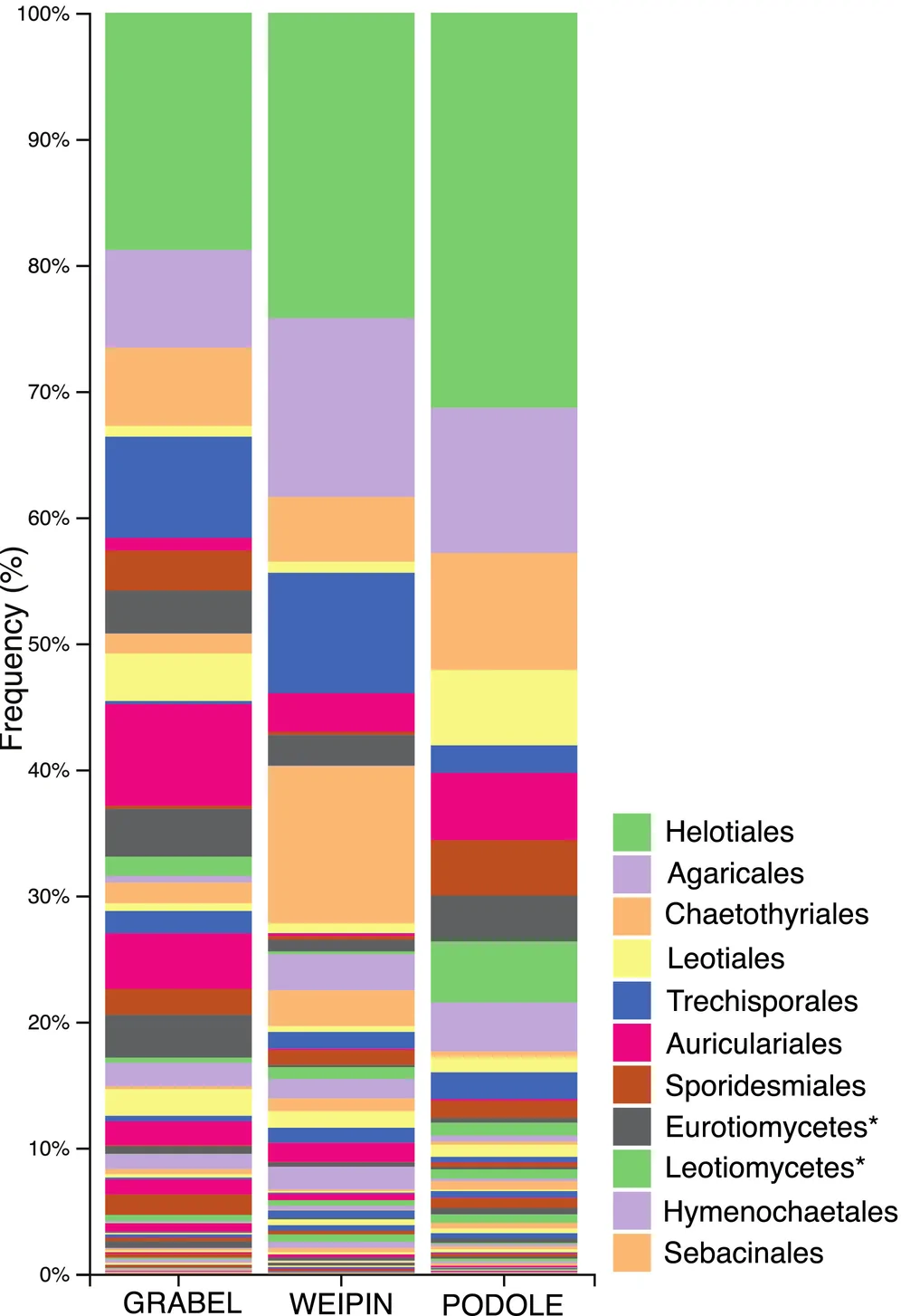
Relative frequency of different orders present in roots of *Graffenrieda* (GRABEL), *Weinmannia* (WEIPIN), and *Podocarpus* (PODOLE). Only the 11 most abundant orders are labeled. *indicates taxa that are *incerta sedis.*﻿﻿

Using the LSU primer set we obtained 264,713 sequence reads, representing 310 OTUs for *Podocarpus*. A separate sequencing run generated 64,511 reads and 442 OTUs for *Graffenrieda* and 57,767 reads and 450 OTUs for *Weinmannia*. Roots of *Graffenrieda* and *Weinmannia* had nearly identical Chao estimated richness (622 and 635 OTUs respectively). After removing OTUs that were present in <4 samples, the final dataset for compositional analyses consisted of 232 OTUs. Host tree species had a significant effect on fungal community composition (Permanova df 2,52, *F* = 5.70, *p* < 0.001; Figure 5), accounting for 18.5% of compositional variation. All three host species significantly differed from each other (Pairwise permanova; *F* > 4.7, *p* < 0.001). Multivariate dispersion differed across host species (df 2,50, *F* = 12.71, *p* < 0.001), with higher dispersion in *Graffenrieda* and *Weinmannia*.

**FIGURE 6:**
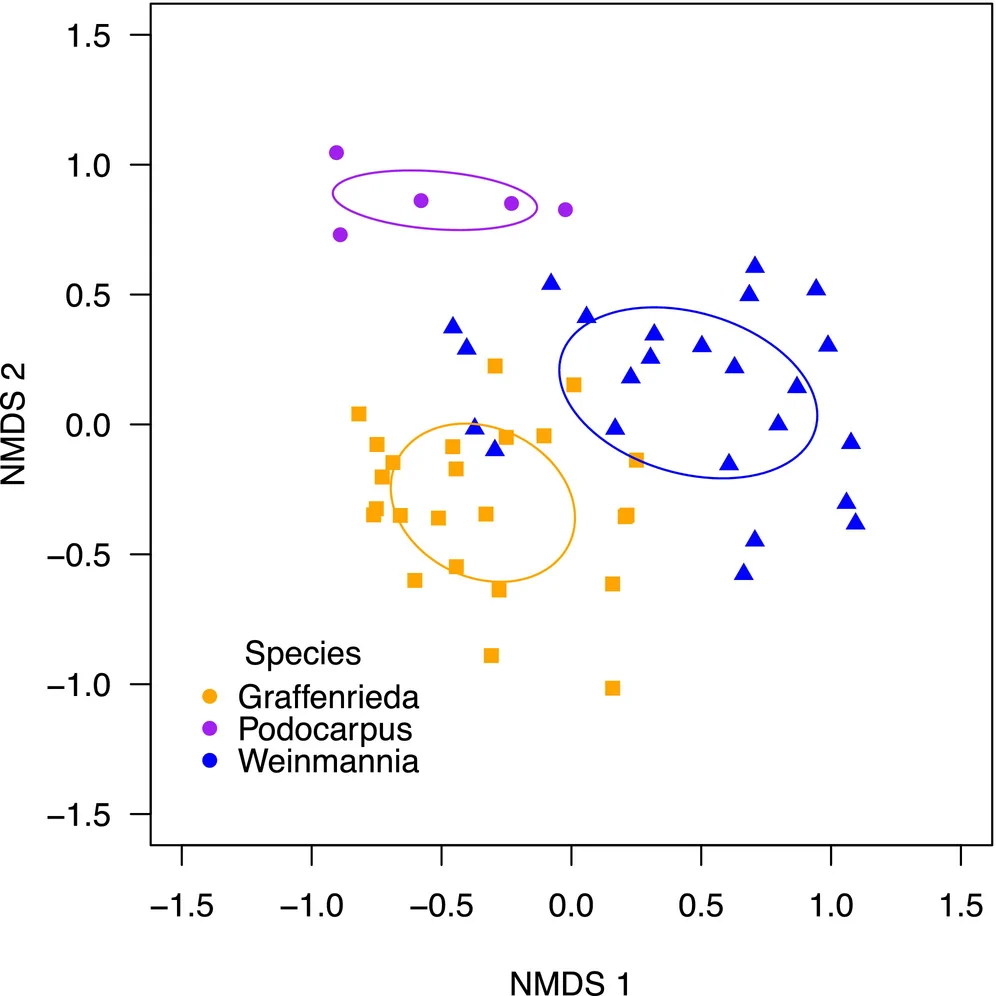
First two axes of a nonmetric multidimensional scaling (NMDS) ordination (*k* = 3, stress = 0.182) representing similarity in fungal communities associated with roots of *Graffenrieda bella*, *Podocarpus oleifolius*, and *Weinmannia pinnata* based on the large subunit (LSU) region. Ellipses represent 1 SD of point scores relative to the species centroid.

**FIGURE 7:**
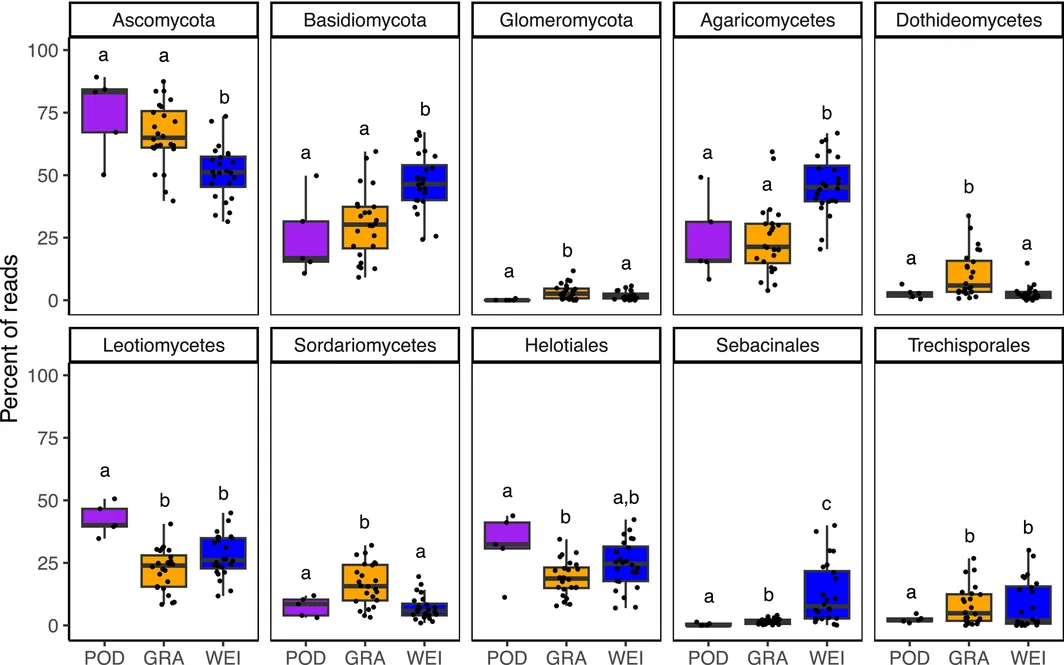
Reads of Ascomycota, Basidomycota, and Glomeromycota expressed as a percent of total reads from the three most abundant phyla; reads of Agaricomycetes, Leotiomycetes, Sordariomycetes, and Dothidiomycetes as a percent of total reads of the four most abundant classes; reads of Helotiales, Trechisporales, and Sebacinales as a percent of total reads of the four most abundant orders. Letters indicate significant differences among species *p* < 0.05. Full model information for species comparisons is provided in Appendix S1: Table S4.

Across the three tree species there were significant differences in the major taxonomic groupings of their root‐associated fungal communities (Figures 6 and 7, Appendix S1: Tables S1–S4). Across phyla, *Podocarpus* had significantly higher Ascomycota and lower Basidiomycota read numbers than *Weinmannia* (Figure 7). Glomeromycota were only detected in very low numbers overall, and were especially low in *Podocarpus* (Figure 7). High Ascomycota read number in *Podocarpus* was reflected in high Leotiomycetes read number, while Sordariomycetes and Dothidiomycetes read numbers were comparatively low (Figure 7). Within Leotiomycetes, read numbers for Helotiales were significantly higher in *Podocarpus* than *Graffenrieda* (Figure 7). Trechisporales, which occurred almost exclusively in aerial roots of *Podocarpus* (Figure 3B), were at much lower relative abundance than in the angiosperms, along with the Agaricomycete order Sebacinales, which was only abundant in *Weinmannia* roots (Figure 7).

Among the most common OTUs, a single taxon of *Hyaloscypha* (Helotiales) accounted for ~21% of the LSU reads of *Podocarpus* and was not found in roots of the other two species (Appendix S1: Table S1). A different species of *Hyaloscypha* was common in *Graffenrieda* and *Weinmannia* roots but rare in *Podocarpus*. The most abundant and third most abundant OTUs in *Weinmannia* were also in the Helotiales, and the second most common was in the Sebacinales. For *Graffenrieda*, the most abundant OTU was in the Capnodiales, followed by an OTU in the Septobasidiales (Basidiomycota) and another in the Helotiales.

## DISCUSSION

We found that diverse communities of fungi colonize *Podocarpus* roots, with 310 OTUs found using LSU primers and 1384 using ITS. While OTU diversity did not differ across microsites, total OTU richness was higher in roots on the litter surface than in the soil. We also found significant differences in community composition among root microsites. While all *Podocarpus* root communities were dominated by Helotiales, fungi in the Trechisporales were much more abundant in aerial roots. Differences in fungal composition among roots that occupy different microsites open the possibility that these root microbiomes also differ in associated resource uptake patterns, potentially reflecting variation in resource availability across microsite types. However, one measure of nutrient uptake, root phosphomonoesterase activity, did not differ across microsites. Roots of *Podocarpus* collected from the soil also differed at the phylum, class, and order levels from those of two co‐occurring angiosperm taxa. These differences in higher taxa of root‐associated fungi may reflect distinct nutrient uptake mutualisms in *Podocarpus* that facilitate its success on low fertility soils.

### Variation in *Podocarpus* root function and fungal community composition across microsites

A striking feature of *Podocarpus* at Chorro is the presence of large aggregations of roots on the litter surface and of aerial roots that climb apogeotropically on the stems of neighboring trees (i.e., from the soil up into the canopy). Long, sparsely branched fine roots are commonly observed draping the trunks of tree ferns, or on the trunks of palms and other trees, often beneath a layer of moss. The production of these aerial roots is presumably an adaptation to acquire nutrients from stem flow from humus that accumulates in the canopy (Vance & Nadkarni, 1992), particularly given the high epiphyte and hemi‐epiphyte loads at Chorro and neighboring watersheds at Fortuna (Gómez Gónzalez et al., 2021). Unusual root adaptations of other tree species are also observed at the Chorro site, including frequent rooting from leaves or small shoots of Melastomataceae blown out of the canopy, and the production of adventitious roots from the vascular bundles of senescent leaves of the Chorro endemic tree fern, *C. rojasiana* (Dalling et al., 2024).

We predicted that differences in the microsites of *Podocarpus* roots would be associated with differences in root chemistry, phosphatase activity, and fungal community composition. In general, aerial roots tended to have lower concentrations of base cations (notably Al and Ca) than roots in soil or on litter, potentially reflecting reduced passive uptake of these elements where they are at lower concentration. In contrast, root P concentration and potential phosphatase activity did not differ across root microsites. Fungal diversity did not differ among root microsites, but richness was highest for litter roots: the intermediate microsite. As predicted, we found fungal communities significantly differed among all three microsites. However, at higher taxonomic levels, the only difference was the abundance of Trechisporales, which accounted for up to 48% of the reads of the four most common orders in aerial roots, but only up to 0.3% of reads for soil roots. Trechisporales, in the class Agricomycetes, consist of a single family (Hydnodontaceae). While species in this group have primarily been characterized as wood and litter decomposers, N isotope data and sequencing from root tips in Brazil and French Guiana suggest a biotrophic, potentially ectomycorrhizal ecology (Vanegas‐León et al., 2019).

The presence of aerial and terrestrial roots is more common in hemi‐epiphytic plants. For vanilla orchids (*Vanilla planifolia*) in Mexico, which harbor both orchid and ectomycorrhizal fungi, Illumina sequencing of fungal ITS also found a significant separation of aerial and terrestrial fungal communities, where aerial roots had greater OTU richness than terrestrial ones (Johnson et al., 2021). Overlap in fungal communities was much greater for orchids: aerial and terrestrial roots shared 86% of OTUs (clustered at 95% sequence similarity), whereas in this study 25% of OTUs classified with ITS were shared. Comparisons of fungal communities of adventitious roots that colonize canopy soil, versus terrestrial roots of *Acer macrophyllum* in temperate rain forests of the Pacific northwest USA (Mafune et al., 2023), also show similar results; fungal richness and diversity measures were lower for canopy soils and community composition was significantly different, while many taxa were shared. Interestingly, multiple genera of dark septate endophytes in the Helotiales were indicator taxa for canopy soils (Mafune et al., 2023). Few comparable studies exist for free‐standing tropical trees. However, the EM canopy tree, *Dicymbe corymbosa*, grows adventitious roots into the mounds of litter and roots that accumulate at the base of the tree and can reach several meters in height. Comparison of the EM‐dominated fungal communities between this organic mound soil and the surrounding mineral soil layer showed similar fungal diversity, but significant compositional differentiation between these rooting habitats (Smith et al., 2017).

### Molecular‐based determination of *Podocarpus* mycorrhizal status

Despite their unusual morphology, very few fungal sequencing studies have examined roots of Podocarpaceae. The first sequencing study, using primers designed to amplify AM fungi, specifically focused on nodules excised from roots of four Podocarpaceae species endemic to New Zealand (Russell et al., 2002). They obtained Glomalean ribosomal DNA sequences from 15 out of 20 nodules, and non‐Glomalean (ascomycete) sequence from two nodules. While the study noted that many taxa were previously unreported, a later blast of their sequence data showed high shared identity with sequences deposited in GenBank, and with no evidence of specificity of AM fungi to Podocarpaceae (Dickie & Holdaway, 2011). A similar sequencing approach was applied to Podocarpaceae roots in Afromontane forest (Wubet et al., 2006). They found comparable diversity and distribution of taxa across the Glomerales phylogeny to those from New Zealand, with sequences mapping to clades representing a range of biomes and plant types. However, while AM fungi showed no evidence of host preference from sequence databases, at the same time, the AM fungi community sequenced from the focal taxon, *Podocarpus falcatus*, was distinct from that of a co‐occurring conifer tree, *Juniperus procera*, sampled at the same sites. Local host preference in AM fungi, resulting in distinct podocarp‐associated AM fungal communities, has been interpreted as potentially reflecting plant–soil feedback effects that influence soil chemistry, and consequently shape local fungal community composition (Dickie & Holdaway, 2011).

Only one previous characterization of Podocarpaceae root fungi exists using high throughput sequencing. Ushio et al. (2017) used Illumina MiSeq to sequence the ITS1 region of seedling roots of 14 tree species from a montane forest at 1500 m elevation at Kinabalu National Park, Malaysia. Their study included five conifer taxa with root nodules (four Podocarpaceae and one in the sister family Araucariaceae). They found 10–40 OTUs per sample, based on clustering with 97% similarity, with approximately equal representation of AM and non‐AM fungal taxa, and no significant difference in OTU richness between conifer and angiosperm roots. In common with the earlier Sanger sequencing studies from New Zealand and Ethiopia, ordination of the Malaysian fungal dataset also showed that podocarps as a group clearly differed in both their AM or non‐AM fungal community composition from that of co‐occurring angiosperms. Within the conifer group, however, there was no clear separation of fungal taxa by host species (Ushio et al., 2017).

The major contrast between these previous sequencing studies and the present study relates to the abundance and diversity of AM fungal OTUs. Ushio et al. (2017) found that AM taxa accounted for a substantial fraction of ITS reads for Malaysian podocarps (mean 4.0%), similar to co‐occurring AM associated angiosperms (mean 5.0%). Furthermore, stained sections of nodule tissue from two of their taxa (*Dacrydium* and *Dacrycarpus*) showed that cells were heavily colonized by arbuscule‐like structures. In contrast, here were found that fungal infection rates were high, while arbuscule‐like structures were relatively uncommon in nodule tissue, and sequencing of ITS showed that only a very small fraction (mean 0.14%) of reads belonged to AM taxa in the Glomerales (Figure 7). Low AM taxon read number may indicate sequencing bias, where other fungal taxa present in a DNA pool preferentially amplify (Tedersoo et al., 2015). However, co‐occurring angiosperm taxa in the same sequencing run had higher numbers of Glomerales reads (1.8%–3.3%). Furthermore, our *Podocarpus* sequencing run also included DNA extractions from the roots of seedlings of pioneer species from lowland Panama. The lowland pioneers had mean Glomerales read fractions of 20% (Ferrer et al., unpublished data), indicating that sequencing bias is unlikely to account entirely for the low abundance of AM fungi of *Podocarpus* in this study, or that montane taxa have higher infection rates from non‐AM root‐associated communities.

### Comparison of *Podocarpus* root mycobiomes with those of co‐occurring angiosperms

Low AM fungal representation and correspondingly high representation of ascomycete fungi appear to be a feature of the root mycobiome of *Podocarpus* from Chorro. In comparison with the two angiosperm taxa, we found a high representation of Leotiomycetes fungi, predominantly in the order Helotiales, and low read abundance of Basidomycota. Leotiomycetes fungi are common root endophytes on coniferous and ericaceous roots in boreal and arctic ecosystems, and in acidic oligotrophic pine barrens (Luo et al., 2017). A recent review has highlighted the Helotiales as an understudied lineage associated with nutrient acquisition across a wide range of host plant taxa (Bruyant et al., 2024). Notably, taxa within this order form a range of associations with host roots. This includes ericoid and ectomycorrhizal associations as well as root endophytic associations. These endophytic associates may promote growth and/or transfer nutrients to hosts even in the absence of conspicuous apoplastic hyphal structures (e.g., Hill et al., 2019; Upson et al., 2009), or may mineralize organic matter releasing inorganic N or P for uptake by co‐infecting AM fungi (Della Monica et al., 2015).

It has been suggested that nutrient transfer from Leotiomycetes fungi is particularly important in areas where AM symbioses are absent (Bruyant et al., 2024). This includes arctic and alpine low fertility environments where plants that form ericoid mycorrhizal symbioses are often abundant (Read, 1991). Although AM fungal taxa were recovered from podocarp roots, low pH, high aluminium saturation, and low availability of mineral nutrients in these forests year‐round may create conditions favoring much greater colonization by Leotiomycetes fungi with correspondingly higher enzymatic diversity and organic matter mineralization capacity. Future work is needed to determine whether Leotiomycetes infection of *Podocarpus* roots is a general feature across the range of habitats occupied by this genus, or is specific to nutrient‐poor montane forests.

Among *Podocarpus* roots sequenced, a single OTU of *Hyaloscypha* (Helotiales) dominated and accounted for ~21% of reads (Appendix S1: Table S1). Despite its abundance in *Podocarpus*, this OTU was not sequenced from the other two taxa. Other Helotiales were also distributed across plant taxa. For example, OTU656 was common in *Weinmannia* and *Graffenrieda* and was also present in low abundance in *Podocarpus* (Appendix S1: Tables S1–S3). Previous work has also highlighted the presence of *Hymenoscyphus* sequences in a complex close to *Hymenoscyphus* *ericae* in the roots of all five trees sequenced of a different *Graffenrieda* species, *G. emarginata*, in nutrient‐poor montane forest in Ecuador (Haug et al., 2011). Microscopy of *G. emarginata* roots also revealed the presence of a hartig net‐like structure, partly or totally covering the fine roots (Haug et al., 2011). A similar hartig net‐like structure also occurs in another species of Cunoniaceae (the family of *Weinmannia*) in rain forest in Australia (McGee & Furby, 1992). In contrast, Haug et al. (2011) report that none of the other 115 tree species that they sequenced from Ecuadorean montane forest formed a similar frequent association with *H. ericae*, and that Hartig net‐like structures were absent except for small patches of roots of two species: our focal study species, *P. oleifolius*, and *Alzatea verticillata* (Alzateaceae), which also occurs at Chorro. Common tree species at the nutrient‐poor Chorro site, including our two angiosperm trees, may therefore all harbor unusual biotrophic fungal partners in their roots.

In conclusion, these results point to a low abundance of AM fungi, and a diversity of other root‐associated fungi that are likely to have mycorrhizal properties for trees in low‐fertility montane forests. The range of potential biotrophic partners results in distinct root‐associated communities across host species, even at quite high taxonomic levels. While many of the potentially beneficial partners belong to Leotiomycetes, other plant groups that are likely mycorrhizal are also present. *Weinmannia* roots had a significantly higher read fraction of Sebacinales than the other two tree species, including its second most abundant OTU (Appendix S1: Table S3). This group is commonly obligate biotrophs that form mycorrhizal associations often with orchids (Weiß et al., 2016). Exploration of the full diversity of root‐associated fungi in tropical montane forests may therefore reveal that mycorrhizal associations in this biome are much more complex than a simple EM‐AM dichotomy.

## AUTHOR CONTRIBUTIONS

Astrid Ferrer, Adriana Corrales, and James W. Dalling conceived the ideas and designed methodology. All authors collected the data. Astrid Ferrer and James W. Dalling analyzed the data and led the writing of the manuscript. Blaine Martin generated confocal microscope images. All authors contributed critically to the drafts and gave final approval for publication.

## CONFLICT OF INTEREST STATEMENT

The authors declare no conflicts of interest.

## Supporting information


Appendix S1:

## Data Availability

All research data, including sequence data information and root nutrient and phosphatase data, are available as follows: Illinois Data Bank in Ferrer et al. (2026) at https://doi.org/10.13012/B2IDB-9955070_V1; National Center for Biotechnology Information (NCBI) Bioproject database under accession number PRJNA1372499 at https://www.ncbi.nlm.nih.gov/bioproject/PRJNA1372499; NCBI Sequence Read Archive database (https://www.ncbi.nlm.nih.gov/sra) under ITS sequence accession numbers SRR36939469–SRR36939512 and LSU accession numbers SRR36940504–SRR36940600.
