# Supplementary material for: Distinct root‐associated fungal communities may account for the persistence of Podocarpus conifers in tropical forest
Source: Ecology. 2026 Sep 25;107(9):e70519. doi: 10.1002/ecy.70519 (PMC13613075; doi:10.1002/ecy.70519)
Supplement: Supplementary file 1 [file ECY-107-e70519-s001.pdf]

Appendix S1

Distinct root-associated fungal communities may account for the persistence of *Podocarpus* conifers in tropical forest

Astrid Ferrer, Blaine Martin, Adriana Corrales, James W Dalling

Ecology

Root staining and confocal microscopy

Fine roots of *Podocarpus* were prepared for confocal microscopy to visualize fungal symbionts and root structures using a combination of fluorescent stains. Roots were first rinsed 3x in water then once gently in phosphate-buffered saline (PBS) to remove soil particles and organic debris. To improve stain penetration and optical clarity, roots were cleared in 10% (w/v) KOH at 95°C in repeated heating and cooling cycles until tissues became tan to pale yellow. Cleared roots were then rinsed several times in PBS before staining.

Wheat Germ Agglutinin conjugated with Alexa Fluor 488 (WGA-AF488) was used to label fungal hyphae and chitin-rich structures. A stock solution was prepared by dissolving 10 mg of WGA-AF488 in 1 mL of PBS. For staining, 10 µL of this stock was diluted in 1 mL of PBS to create a working solution. Roots were incubated overnight at room temperature in the dark, then rinsed several times with PBS to remove excess stain.

Following WGA staining, roots were counterstained with Propidium Iodide (PI) to highlight cell walls and host tissue boundaries. A stock solution was prepared by dissolving 10 mg of PI (>94% HPLC) in 1 mL of PBS. The working solution consisted of 10 µL of PI stock in 1 mL of PBS. Roots were incubated in the PI working solution for 30 minutes at room temperature, protected from light, and rinsed several times in PBS.

Stained roots were mounted in a glycerol-based solution of PBS and imaged using a Zeiss LSM 880 Airyscan confocal microscope at the Institute for Genomic Biology Core Facility at the University of Illinois, Urbana-Champaign. Images were collected using a range of optical settings, including z-stacks, tile scans, and variable laser intensities, to capture both surface and internal fungal structures across root tissues. Excitation and emission settings were adjusted for each fluorophore (WGA-AF488: 488/500–550 nm; PI: 535/617 nm).

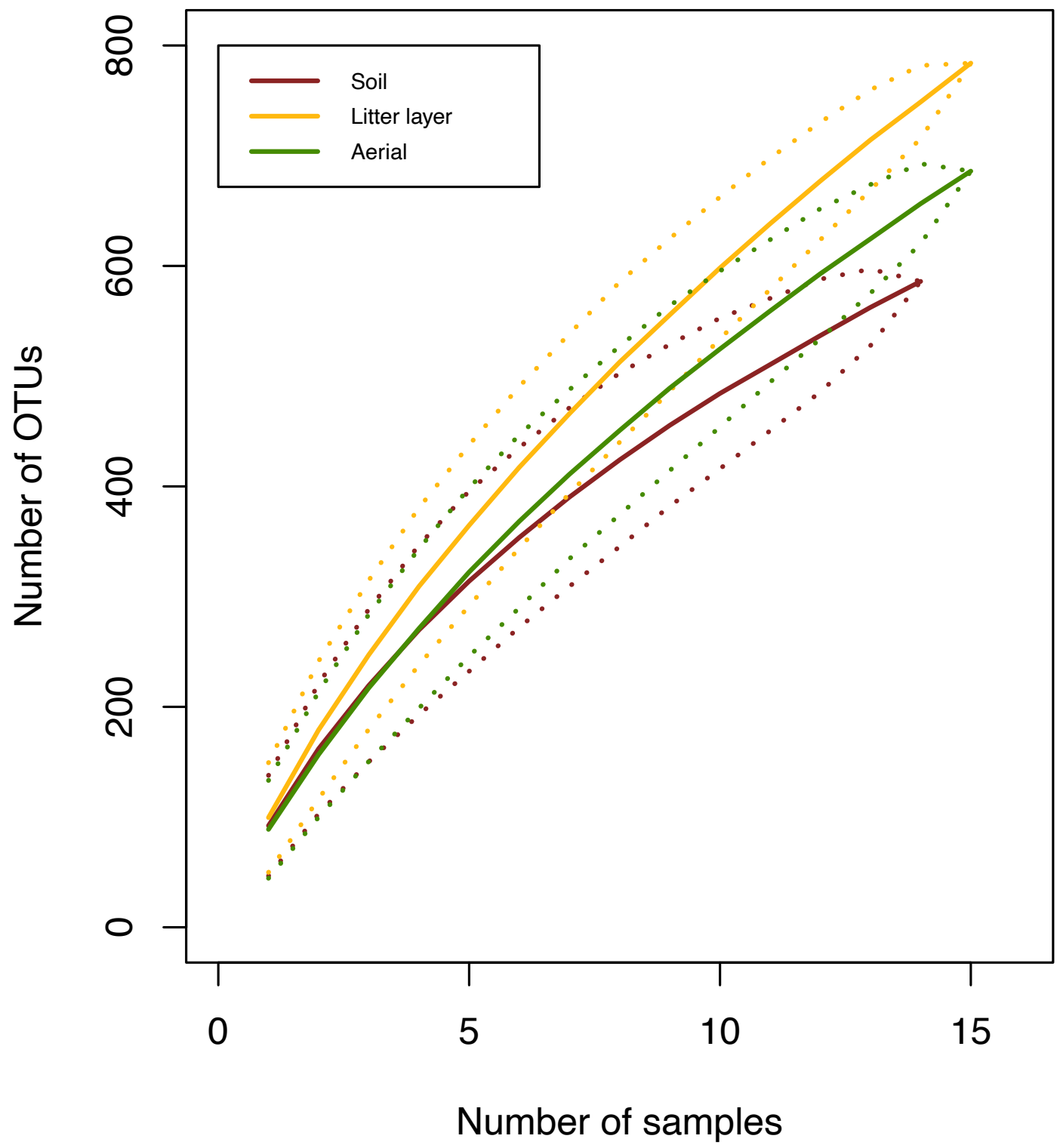

Figure S1: Fungal OTU accumulation curves derived from ITS data for three root microsites (soil, litter and aerial microsites) sampled at five *Podocarpus oleifolius* trees.

Table S1: The 30 most abundant OTUs (by read abundance) found in roots excavated from soil of *Podocarpus oleifolius* based on sequencing of the fungal ribosomal large subunit (LSU) using the primers LR0R-LR3. Percent reads represents percent of all reads for *Podocarpus* = 264,713 reads. The top 30 OTUs account for 81.2% of all reads.

OTU number	Percent reads	*Taxonomy*
OTU324	20.9	Ascomycota_Leotiomycetes_Helotiales_Hyaloscyphaceae_Hyaloscypha
OTU759	5.1	Basidiomycota_Agaricomycetes_Auriculariales_Hyaloriaceae_Bourdotia
OTU846	5.0	Ascomycota_Eurotiomycetes_Chaetothyriales_Herpotrichiellaceae_Capronia
OTU589	4.4	Ascomycota_Sordariomycetes_Sporidesmiales_Sporidesmiaceae_Sporidesmium
OTU857	3.8	Basidiomycota_Agaricomycetes_Hymenochaetales
OTU609	3.6	Ascomycota_Leotiomycetes_Helotiales_Hyaloscyphaceae
OTU1491	3.6	Ascomycota_Leotiomycetes_Leotiales_Tympanidaceae_Claussenomyces
OTU1615	3.3	Basidiomycota_Agaricomycetes_Agaricales_Entolomataceae_Entoloma
OTU384	2.9	Ascomycota_Eurotiomycetes
OTU95	2.7	Ascomycota_Leotiomycetes_Leotiomycetes_inc._sedis_Myxotrichaceae_Oidiodendron
OTU380	2.5	Ascomycota_Eurotiomycetes_Chaetothyriales_Herpotrichiellaceae_Cladophialophora
OTU896	2.4	Ascomycota_Leotiomycetes_Helotiales
OTU1214	2.4	Basidiomycota_Agaricomycetes_Agaricales_Marasmiaceae_Marasmius
OTU138	2.3	Ascomycota_Leotiomycetes_Leotiales_Tympanidaceae_Claussenomyces
OTU2575	2.1	Ascomycota_Lecanoromycetes_Ostropales_Stictidaceae_Cryptodiscus
OTU1912	2.1	Ascomycota_Leotiomycetes_Leotiomycetes_inc._sedis_Myxotrichaceae_Oidiodendron
OTU996	1.6	Basidiomycota_Agaricomycetes_Agaricales_Entolomataceae_Richoniella
OTU2949	1.5	Basidiomycota_Agaricomycetes_Agaricales_Tricholomataceae_Mycena
OTU624	1.4	Basidiomycota_Agaricomycetes_Agaricales_Clavariaceae_Clavulinopsis
OTU2075	1.1	Ascomycota_Leotiomycetes_Helotiales
OTU3191	1.0	Ascomycota
OTU15	0.8	Ascomycota_Dothideomycetes
OTU2408	0.8	Ascomycota_Leotiomycetes_Helotiales_Hyaloscyphaceae_Hyaloscypha
OTU69	0.8	Ascomycota_Dothideomycetes_Venturiales_Venturiaceae_Apiosporina
OTU109	0.7	Ascomycota_Leotiomycetes_Helotiales
OTU16	0.7	Basidiomycota_Agaricomycetes_Trechisporales_Trech._inc._sedis_Sistotremastrum
OTU825	0.5	Basidiomycota_Agaricomycetes_Gomphales_Gomphaceae_Ramaria
OTU2830	0.5	Basidiomycota_Agaricomycetes_Polyporales_Fomitopsidaceae_Antrodia
OTU882	0.4	Ascomycota_Eurotiomycetes_Sclerococcales_Dactylosporaceae_Fusichalara
OTU834	0.4	Ascomycota_Sordariomycetes_Hypocreales

Table S2: The 30 most abundant OTUs (by read abundance) found in roots excavated from soil of *Graffenrieda bella* based on sequencing of the fungal ribosomal large subunit (LSU) using the primers LR0R-LR3. Percent reads represents percent of all reads for *Graffenrieda* =64,511 reads. The top 30 OTUs account for 64.7% of all reads.

OTU number	Percent reads	*Taxonomy*
OTU1568	8.0	Ascomycota_Dothideomycetes_Capnodiales_Penidiella
OTU440	4.4	Basidiomycota_Pucciniomycetes_Septobasidiales_Septobasidiaceae_Septobasidium
OTU1072	3.9	Ascomycota_Leotiomycetes_Helotiales_Helotiaceae_Hymenoscyphus
OTU656	3.4	Ascomycota_Leotiomycetes_Helotiales_Hyaloscyphaceae_Hyaloscypha
OTU824	3.3	Basidiomycota_Agaricomycetes_Trechisporales
OTU1214	2.9	Basidiomycota_Agaricomycetes_Agaricales_Marasmiaceae_Marasmius
OTU635	2.8	Ascomycota_Sordariomycetes
OTU3052	2.8	Ascomycota_Leotiomycetes_Helotiales_Scytalidium
OTU774	2.8	Ascomycota_Sordariomycetes_Sordariales
OTU609	2.5	Ascomycota_Leotiomycetes_Helotiales_Hyaloscyphaceae
OTU589	2.4	Ascomycota_Sordariomycetes_Sporidesmiales_Sporidesmiaceae_Sporidesmium
OTU834	2.1	Ascomycota_Sordariomycetes_Hypocreales
OTU2001	1.8	Ascomycota_Leotiomycetes_Helotiales_Hyaloscyphaceae_Hyalopeziza
OTU110	1.7	Ascomycota_Lecanoromycetes
OTU703	1.7	Basidiomycota_Agaricomycetes_Corticiales_Corticiaceae_Sistotrema
OTU915	1.7	Ascomycota_Eurotiomycetes_Chaetothyriales_Herpotrichiellaceae_Cladophialophora
OTU112	1.6	Ascomycota_Eurotiomycetes_Chaetothyriales_Herpotrichiellaceae_Phialophora
OTU2932	1.6	Basidiomycota_Pucciniomycetes_Helicobasidiales_Helicobasidiaceae_Tuberculina
OTU2281	1.4	Basidiomycota_Agaricomycetes_Cantharellales_Hydnaceae_Clavulina
OTU275	1.4	Ascomycota_Leotiomycetes_Helotiales_Hyaloscyphaceae
OTU39	1.4	Basidiomycota_Agaricomycetes_Trechisporales_Hydnodontaceae_Luellia
OTU205	1.3	Ascomycota_Sordariomycetes_Hypocreales
OTU929	1.1	Basidiomycota_Agaricomycetes_Agaricales_Bolbitiaceae_Bolbitius
OTU1338	1.1	Basidiomycota_Agaricomycetes_Trechisporales_Hydnodontaceae_Trechispora
OTU623	1.0	Ascomycota_Eurotiomycetes
OTU851	1.0	Basidiomycota_Agaricomycetes_Boletales_Boletaceae_Neoboletus
OTU1807	1.0	Ascomycota_Orbiliomycetes_Orbiliales_Orbiliaceae_Orbilia
OTU256	1.0	Basidiomycota_Agaricomycetes_Agaricales_Porotheleaceae_Pseudohydropus
OTU2144	1.0	Ascomycota_Leotiomycetes
OTU807	0.8	Ascomycota_Leotiomycetes_Leotiales_Leotiaceae_Alatospora

Table S3: The 30 most abundant OTUs (by read abundance) found in roots excavated from soil of *Weinmannia pinnata* based on sequencing of the fungal ribosomal large subunit (LSU) using the primers LR0R-LR3. Percent reads represents percent of all reads for *Weinmannia* = 57,767 reads. The top 30 OTUs account for 63.5% of all reads.

OTU number	Percent reads	*Taxonomy*
OTU275	8.4	Ascomycota_Leotiomycetes_Helotiales_Hyaloscyphaceae
OTU1	8.0	Basidiomycota_Agaricomycetes_Sebacinales_Sebacinaceae
OTU2001	4.4	Ascomycota_Leotiomycetes_Helotiales_Hyaloscyphaceae_Hyalopeziza
OTU929	3.7	Basidiomycota_Agaricomycetes_Agaricales_Bolbitiaceae_Bolbitius
OTU1874	3.5	Basidiomycota_Agaricomycetes_Trechisporales_Hydnodontaceae_Luellia
OTU1739	3.4	Basidiomycota_Agaricomycetes_Trechisporales_Hydnodontaceae_Subulicystidium
OTU838	2.6	Ascomycota_Leotiomycetes
OTU656	2.5	Ascomycota_Leotiomycetes_Helotiales_Hyaloscyphaceae_Hyaloscypha
OTU609	2.0	Ascomycota_Leotiomycetes_Helotiales_Hyaloscyphaceae
OTU112	2.0	Ascomycota_Eurotiomycetes_Chaetothyriales_Herpotrichiellaceae_Phialophora
OTU787	2.0	Basidiomycota_Agaricomycetes_Agaricales_Tricholomataceae_Mycena
OTU416	1.8	Ascomycota_Sordariomycetes_Diaporthales_Sydowiellaceae_Calosporella
OTU1707	1.7	Basidiomycota_Agaricomycetes_Sebacinales_Serendipitaceae_Serendipita
OTU664	1.7	Basidiomycota_Agaricomycetes_Agaricales_Schizophyllaceae_Henningsomyces
OTU110	1.5	Ascomycota_Lecanoromycetes
OTU644	1.3	Basidiomycota_Agaricomycetes_Auriculariales_Hyaloriaceae_Bourdotia
OTU851	1.2	Basidiomycota_Agaricomycetes_Boletales_Boletaceae_Neoboletus
OTU623	1.2	Ascomycota_Eurotiomycetes
OTU888	1.2	Ascomycota_Eurotiomycetes_Chaetothyriales_Herpotrichiellaceae
OTU1013	1.2	Ascomycota_Leotiomycetes_Helotiales
OTU458	1.0	Basidiomycota_Agaricomycetes_Agaricales_Tricholomataceae_Mycena
OTU39	1.0	Basidiomycota_Agaricomycetes_Trechisporales_Hydnodontaceae_Luellia
OTU344	0.9	Basidiomycota_Agaricomycetes_Sebacinales_Serendipitaceae_Serendipita
OTU3327	0.9	Ascomycota_Leotiomycetes_Helotiales_Bryoglossaceae_Bryoclaviculus
OTU807	0.9	Ascomycota_Leotiomycetes_Leotiales_Leotiaceae_Alatospora
OTU1121	0.8	Basidiomycota_Agaricomycetes_Agaricales_Tricholomataceae_Mycena
OTU1550	0.8	Ascomycota_Leotiomycetes_Helotiales__Nagrajchalara
OTU410	0.8	Basidiomycota_Agaricomycetes_Agaricales_Mycenaceae_Resinomycena
OTU2232	0.7	Basidiomycota_Agaricomycetes_Trechisporales_Hydnodontaceae_Trechispora
OTU3052	0.7	Ascomycota_Leotiomycetes_Helotiales__Scytalidium

Table S4: Model output for species effects on the proportion of sequence reads belonging to each phylum, class and order shown in Figure 7. The first line shows the overall model significance. Species comparisons show adjusted p values from Tukey's HSD for gaussian models. Quasi-binomial error models were used in cases where gaussian errors did not meet model assumptions (Glomeromycota, Dothidiomycetes, Sebacinales and Trechisporales).

Phylum Ascomycota	df 2,50; $F=13.61$, $p<0.001$
Graffenrieda vs Podocarpus	$p=0.32$
Weinmannia vs Podocarpus	$p<0.001$
Graffenrieda vs Weinmannia	$p<0.001$
Phylum Basidiomycota	df 2,50; $F=12.78$, $p<0.001$
Graffenrieda vs Podocarpus	$p=0.66$
Weinmannia vs Podocarpus	$p<0.001$
Graffenrieda vs Weinmannia	$p<0.001$
Phylum Glomeromycota	df 2,50; $\chi^2=71.70$, $p<0.001$
Graffenrieda vs Podocarpus	$p<0.001$
Weinmannia vs Podocarpus	$p<0.001$
Graffenrieda vs Weinmannia	$p=0.08$
Class Agaricomycetes	df 2,50; $F=17.89$, $p<0.001$
Graffenrieda vs Podocarpus	$p=0.32$
Weinmannia vs Podocarpus	$p=0.32$
Graffenrieda vs Weinmannia	$p=0.32$
Class Dothidiomycetes	df 2,50; $\chi^2=24.39$, $p<0.001$
Graffenrieda vs Podocarpus	$p<0.001$
Weinmannia vs Podocarpus	$p=0.92$
Graffenrieda vs Weinmannia	$p=0.003$
Class Leotiomycetes	df 2,50; $F=12.75$, $p<0.001$
Graffenrieda vs Podocarpus	$p<0.001$
Weinmannia vs Podocarpus	$p=0.002$
Graffenrieda vs Weinmannia	$p=0.056$
Class Sordariomycetes	df 2,50; $F=12.89$, $p<0.001$
Graffenrieda vs Podocarpus	$p=0.024$
Weinmannia vs Podocarpus	$p=0.98$
Graffenrieda vs Weinmannia	$p<0.001$
Order Helotiales	df 2,50; $F=5.25$, $p<0.001$
Graffenrieda vs Podocarpus	$p=0.012$
Weinmannia vs Podocarpus	$p=0.20$
Graffenrieda vs Weinmannia	$p=0.10$

Order Sebacinales	df 2,50; χ^2=100.18, p<0.001
Graffenrieda vs Podocarpus	p=0.04
Weinmannia vs Podocarpus	p=0.001
Graffenrieda vs Weinmannia	p<0.001
Order Trechisporales	df 2,50; χ^2=31.8, p<0.001
Graffenrieda vs Podocarpus	p<0.001
Weinmannia vs Podocarpus	p<0.001
Graffenrieda vs Weinmannia	p=0.56
